# Scale effects of nanomechanical properties and deformation behavior of Au nanoparticle and thin film using depth sensing nanoindentation

**DOI:** 10.3762/bjnano.5.94

**Published:** 2014-06-11

**Authors:** Dave Maharaj, Bharat Bhushan

**Affiliations:** 1 Nanoprobe Laboratory for Bio-& Nanotechnology and Biomimetics (NLBB), The Ohio State University, 201 W.19th Avenue, Columbus, Ohio 43210-1142, USA

**Keywords:** gold (Au), Hall–Petch, hardness, nanoindentation, nano-objects

## Abstract

Nanoscale research of bulk solid surfaces, thin films and micro- and nano-objects has shown that mechanical properties are enhanced at smaller scales. Experimental studies that directly compare local with global deformation are lacking. In this research, spherical Au nanoparticles, 500 nm in diameter and 100 nm thick Au films were selected. Nanoindentation (local deformation) and compression tests (global deformation) were performed with a nanoindenter using a sharp Berkovich tip and a flat punch, respectively. Data from nanoindentation studies were compared with bulk to study scale effects. Nanoscale hardness of the film was found to be higher than the nanoparticles with both being higher than bulk. Both nanoparticles and film showed increasing hardness for decreasing penetration depth. For the film, creep and strain rate effects were observed. In comparison of nanoindentation and compression tests, more pop-ins during loading were observed during the nanoindentation of nanoparticles. Repeated compression tests of nanoparticles were performed that showed a strain hardening effect and increased pop-ins during subsequent loads.

## Introduction

The characterization of mechanical properties is crucial for a fundamental understanding of materials behavior during contact. Mechanical properties of interest comprise hardness, Young’s modulus of elasticity, bulk modulus, elastic–plastic deformation, scratch resistance, residual stresses, time-dependent creep and relaxation properties, fracture toughness, fatigue and yield strength.

With the advent of the atomic force microscope (AFM) and specialized commercial depth-sensing indenters, the probing of mechanical properties on the micro- and nanoscale under ultra- low loads has become possible [1–2]. In particular, the use of a nanoindenter with depth sensing is ideal, as mechanical properties such as hardness and Young’s modulus of elasticity can be directly obtained as a function of depth. This can be done with a high degree of accuracy, not easily obtained with an AFM. This advancement in technology has proven useful for understanding the mechanical behavior of micro- and nano-objects that are continually being developed and incorporated into a wide variety of macro- to nanoscale systems [3]. With the depth-sensing nanoindenter, indentation studies with a sharp three-sided pyramidal Berkovich tip and compression studies with a flat punch have been performed. The sharp tip allows for the study of localized deformations and the flat punch allows for the study of deformations of entire micro- or nano-objects. Knowledge of their mechanical properties is crucial for predicting deformation behavior under various loading regimes, which is important for long term use. Research has shown that mechanical properties on the micro- to nanoscale are different from bulk and are scale-dependent as was observed in studies of bulk solid surfaces, surface thin films and micro- to nano-objects. Mechanical properties can either be reduced or enhanced. There are many theories and mechanisms used to explain the state of mechanical properties and deformation behavior of materials on the macro- to nanoscale. Evaluation of each along with experimental conditions is necessary to explain and place new research in context.

Reduced mechanical properties have been observed in some studies of micro/nano-objects where decreasing diameters results in a reduction of yield strength and hardness [4–6]. This has been explained according to the inverse Hall–Petch effect, which means that mechanical properties, below a critical grain size, decreases as grain sizes becomes smaller resulting in reduced properties. Jang and Greer [6], for example reported for Ni micropillars (0.1–2.5 µm) under compression, that the yield strength was reduced as the diameter was decreased with a critical grain size of 60 nm. Of particular interest are cases in which properties are enhanced leading to the ‘smaller is stronger’ phenomenon. An overview of several of these studies, in which mechanical properties improve as scale is reduced, is presented in Table 1. Also presented are material dimensions and associated theories.

**Table 1:** Review of studies of enhanced scale-dependent mechanical properties of bulk solid surfaces, thin films and various nano-objects. For thin films, the thicknesses are given and for nano-objects, the diameters are given. Pillars described in the studies have low aspect ratios (2–20) compared to wires (>20).

material	method	result	theories

solid surfaces			

Ag, Au, Cu, GaAs, GaP, Ni, Si, Ti, ZnSe	indentation [7–13]	hardness higher than bulk; hardness inversely proportional to indentation depth	strain gradient plasticity [29–30]

thin films			

Al (210–1090 nm)	indentation [14]	hardness inversely proportional to film thickness	Hall–Petch [31–34], dislocation constraint [45]
Ag, Au (100–2000 nm)	indentation [15]		Hall–Petch [31–34]
Au (31–858 nm)	indentation [16]		
Ni (50–700 nm)	indentation [17]	hardness higher than bulk	dislocation constraint [45]

nano-objects			

Cu/Nb microwires (1–10 µm)	indentation [18]	hardness inversely proportional to diameter	Hall–Petch [31–34]
Au micropillars (0.4–7.5 µm)	compression [19]	yield stress higher than bulk; yield stress inversely proportional to diameter	dislocation starvation [19]
Au nanowires (40–250 nm)	bending [20]	yield strength inversely proportional to diameter and greater than bulk	Hall–Petch [31–34]
Ni nanopillars (150–400 nm)	compression [21]	yield stress inversely proportional to diameter	dislocation starvation [19]
Au nanopillars (150–1000 nm)	compression [22]	yield strength inversely proportional to diameter	Hall–Petch [31–34]
Au nanoparticles (200–1000 nm)	compression [26]	smaller nanoparticles yield at higher stress	dislocation entanglement [26]

In studies of bulk solid surfaces [7–13] and thin films [14–17] made of various materials including Al, Au, Ag, Cu, GaAs, GaP, Si, Ti, ZnSe and Ni, scale effects on hardness with respect to the depth of penetration or indentation size effect (ISE) and decreasing film thickness have been reported. Pharr and Oliver [9] and Bhushan et al. [11], for example, found that a decreasing indentation depth resulted in higher hardness of Ag and Si surfaces, respectively. In both cases the hardness of the nano-objects was higher than that of the bulk material. Other studies revealed the relationship between film thickness and hardness. Cao et al. [15] and Dietiker et al. [16], for example, demonstrated that as the film thickness decreased for Ag and Au (10–2000 nm) and solely Au (31–858 nm), respectively, hardness increased. The dependence of the hardness on the film thickness can be explained by either the Hall–Petch effect or dislocation constraint, where the hard substrate limits the movement of dislocations. Large strain gradients observed in the ISE also contribute to the material hardness. Scale dependence is also seen in studies of various micro/nano-objects. Enhanced mechanical properties were observed in nano-objects [18–23] made of various materials including Au, Cu, Nb and Ni, for which decreasing diameters result in an increase in micro/nano-object yield stress and hardness. Indentation tests of Cu/Nb microwires (1–10 µm) by Thilly et al. [18] showed that a lower diameter resulted in higher yield stress following the ‘smaller is stronger’ phenomenon. In compression studies involving Au (0.4–7.5 µm) micropillars a similar observation was made and higher yield strengths were observed compared to bulk with decreasing micropillar diameter [19]. For a thorough review, see Palacio and Bhushan [24]. The increase in yield strength or hardness seen with nano-objects has been explained by the dislocation starvation model or the Hall–Petch effect for single crystalline and polycrystalline nano-objects, respectively. In the dislocation starvation model, the absence of dislocations in the interior of the nano-object does not allow for plastic deformation to occur. Similar to thin films, for indentation of micro and nano-objects, there is also a contribution to hardening due to the occurrence of large strain gradients at shallow depths for both single and polycrystalline materials. Details of the mechanisms which lead to enhanced hardness with polycrystalline bulk solids, surface thin films and nano-objects are presented in the next section. These mechanisms are explained to aid in understanding of mechanical properties and deformation behavior of materials.

Nanoparticles made of Au are of interest since they are used in tribological applications on the macro- to nanoscale and applications requiring controlled manipulation and targeting [25]. In these environments the nanoparticles can be deformed locally or the entire nanoparticle can be compressed. Knowledge of the mechanical properties and deformation mechanisms involved when subjected to an applied load is important for determining their suitability for various applications. Studies have been previously performed on Au nanoparticles by doing indentation experiments to look at the effect of lateral dimension (elongation) on strength [26] and by doing compression experiments to study the effect of overall particle size on strength [23]. Studies that directly compare indentation (local deformation) with compression (global deformation) with nanoparticles of the same size and geometry to understand the differences in deformation modes are lacking. In addition to indentation studies of Au nanoparticles, it is of interest to study thin Au films. As the size of the nanoparticle decreases there is less contact with the tip due to the curvature of both the tip and nanoparticle and this can lead to inaccuracies in determining the contact area. This results in errors during obtaining the mechanical properties. Thin films, due to their flat surfaces, eliminate this problem and provide an opportunity to further investigate scale effects of mechanical properties of a material at smaller dimensions. This is due to a more accurate determination of the contact area than would be possible with a nanoparticle of similar size.

In this paper, 500 nm Au nanoparticles and a 100 nm thick Au film were investigated to determine scale effects of mechanical properties and deformation behavior. Various normal loads were applied through nanoindentation with a sharp tip (local deformation) and compression with a flat punch (global deformation). Data from the nanoindentation studies were compared with bulk to study scale effects on hardness. The effects of the penetration depth on hardness were investigated for nanoparticles and thin films. For the films, creep and strain rate tests were also studied. Load effects were compared between loading methods to understand the mechanisms involved during deformation. Repeated compression tests of nanoparticles were performed to study nanoscale strain hardening.

### Mechanisms

In this section, mechanisms for the observed enhanced mechanical properties of polycrystalline materials on the nanoscale are described. Explanations of the various mechanisms are important to aid in discussing and understanding the current research. In some cases one or multiple explanations can account for the observed mechanical properties and deformation behavior. It is necessary to understand the details of these mechanisms to determine which ones apply and best explains the results. As physical dimensions reach the nanoscale, an increase in yield stress or hardening is observed compared to the macroscale. These changes are driven by the presence or absence of sets of atoms that disrupt the regular atomic arrangements in the lattice planes, the so called dislocations.

**Figure 1:**
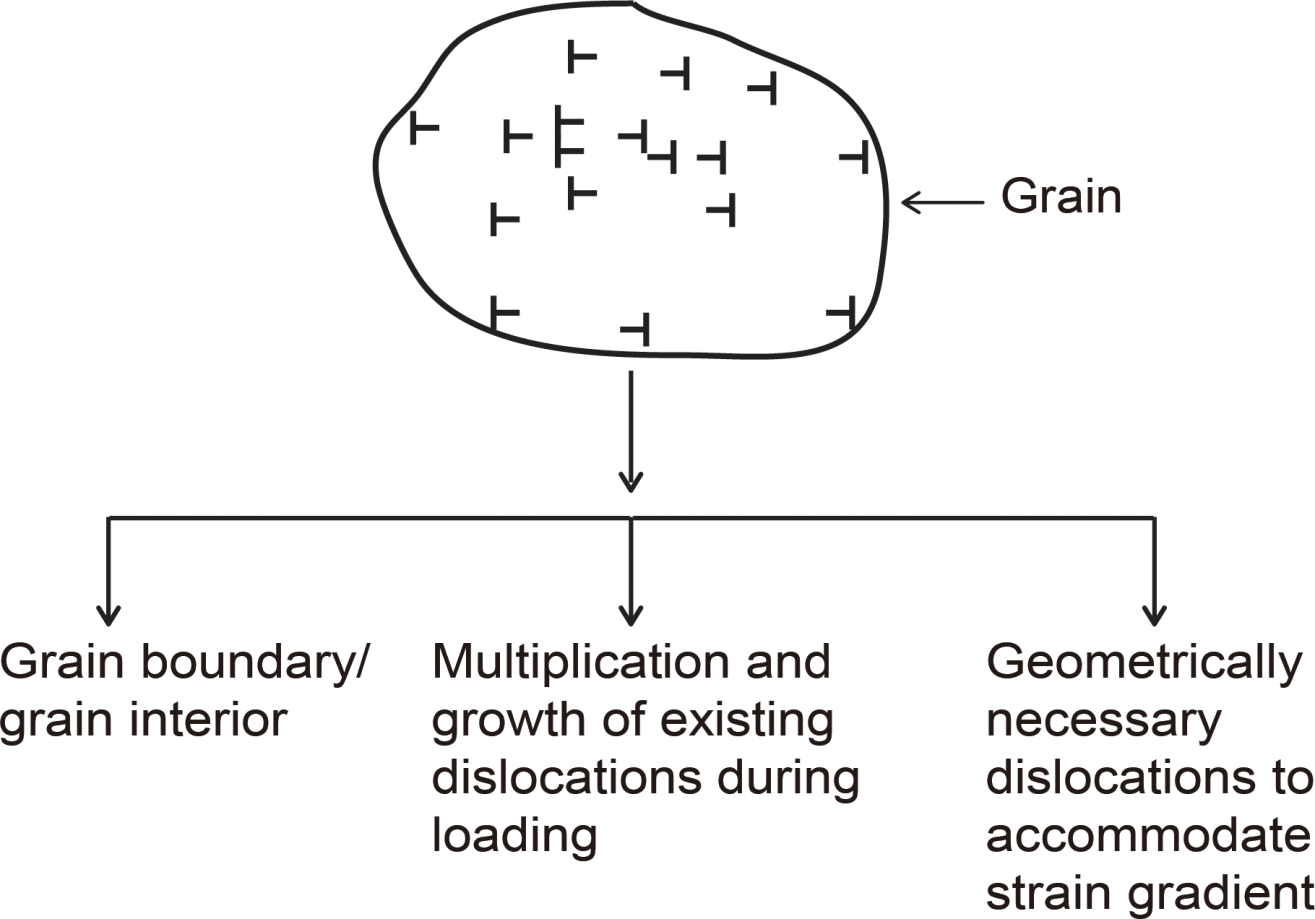
Flowchart illustrating dislocation sources, in a grain, from the grain boundary and the grain interior, from multiplication and growth of an existing dislocation and from geometrically necessary dislocations needed to accommodate strain gradient.

Figure 1 shows for a polycrystalline material, as an example, dislocations in the grain originating from the grain boundary and the grain interior, from a multiplication of existing dislocations during loading or from geometrically necessary dislocations (GNDs) generated to accommodate strain gradient in nanoindentation at low penetration depths.

Movement of dislocations by slip allows for plastic deformation to occur [27]. After an initial slip, as more dislocations are generated there is an increase in yield stress or hardness as the dislocations interact with each other or at the grain boundaries in polycrystalline material. These interactions are responsible for the trend of enhanced mechanical properties with reduction in size or the ‘smaller is stronger’ phenomenon. They help to explain certain observable effects such as the ISE and Hall–Petch effect. The mechanisms of each are given in the following sections. In the case of the ISE, contributions to enhanced hardness can occur in either single or polycrystalline nano-objects.

### Indentation size effect: Strain gradient plasticity

**Figure 2:**
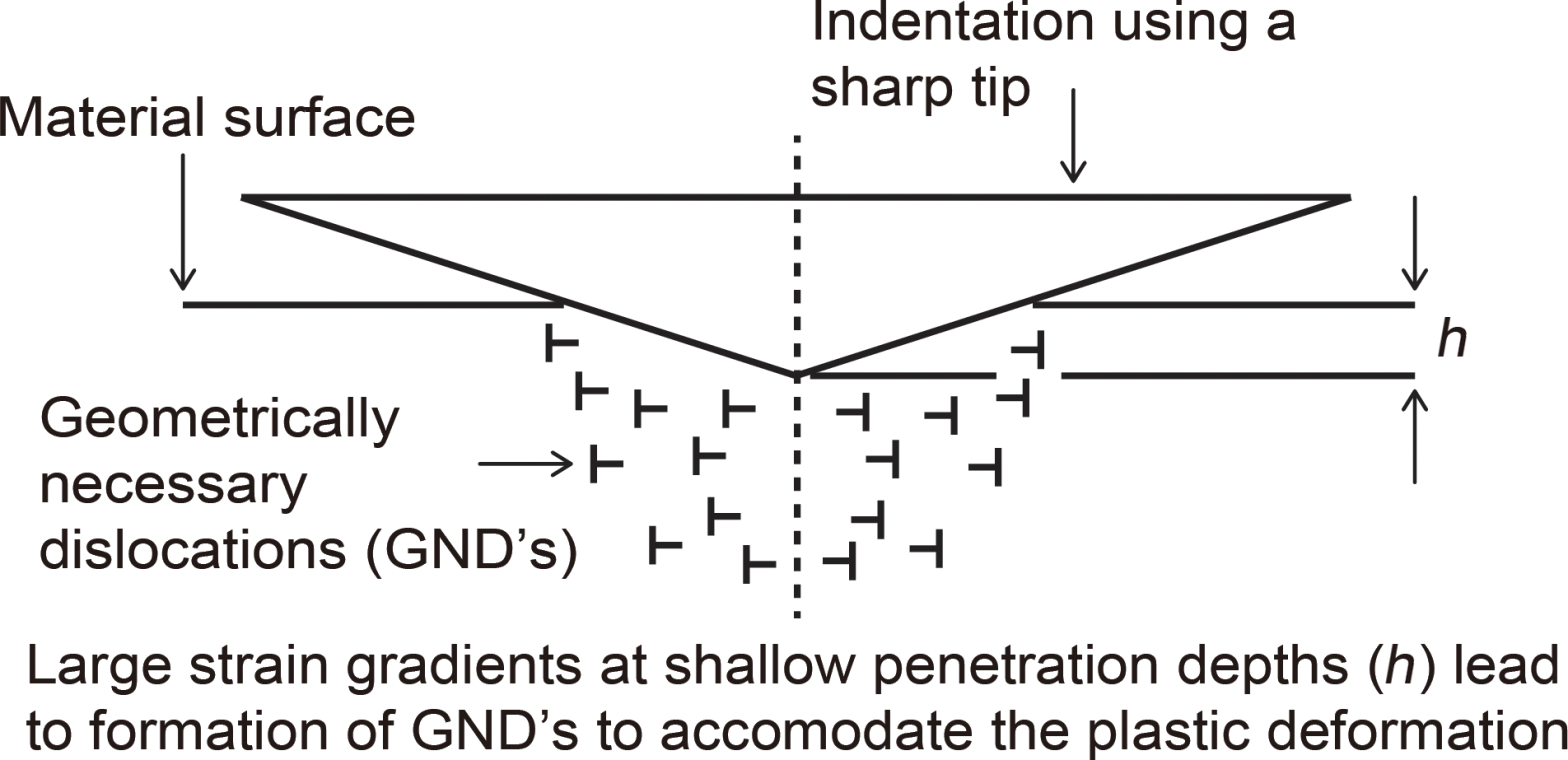
Illustration of the strain gradient plasticity theory in which high strain gradients occur at shallow indentation depths with a sharp tip. This leads to geometrically necessary dislocations (GNDs) being generated to accommodate for the strain gradient and a subsequent permanent deformation. The GNDs entangle and impede further movement of existing dislocations as well as the formation and movement of new dislocations.

Indentation of materials with a sharp tip at shallow depths leads to large strain gradients, which results in formation of GNDs. This allows for the accommodation of plastic deformation of the material beneath the indenter, as depicted in Figure 2. The GNDs along with dislocations which are formed in the absence of strain gradients, known as statistically stored dislocations (SSD), hinder the formation and movement of new dislocations. As indentation depths decreases larger strain gradients lead to an increase in the density of dislocations. This results in a strengthening effect [28–30] and accounts for the observed increase in hardness at shallower indentation depths. This phenomenon was first modeled by Nix and Gao [30] according to the following relation

[1]
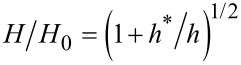


where *H* is the hardness at a given indentation depth *h*, with *H*_0_ being the hardness at a large indentation depth and h^*^ is a characteristic length, which is dependent on the indenter shape, the shear modulus and *H*_0_. The ISE contributes to increased hardness in bulk solid surfaces, thin films and nano-objects.

### Hall–Petch effect

The generation of dislocations leads to the Hall–Petch effect through the dislocation pile up mechanism or the dislocation density mechanism and are described in subsequent sections. In both mechanisms as the grain size is reduced, the yield stress increases resulting in higher hardness in the case of indentation as stated previously [31–34]. It should be noted that the strengthening effect can also be the result of a combination of mechanisms.

#### Dislocation pile-up mechanism

**Figure 3:**
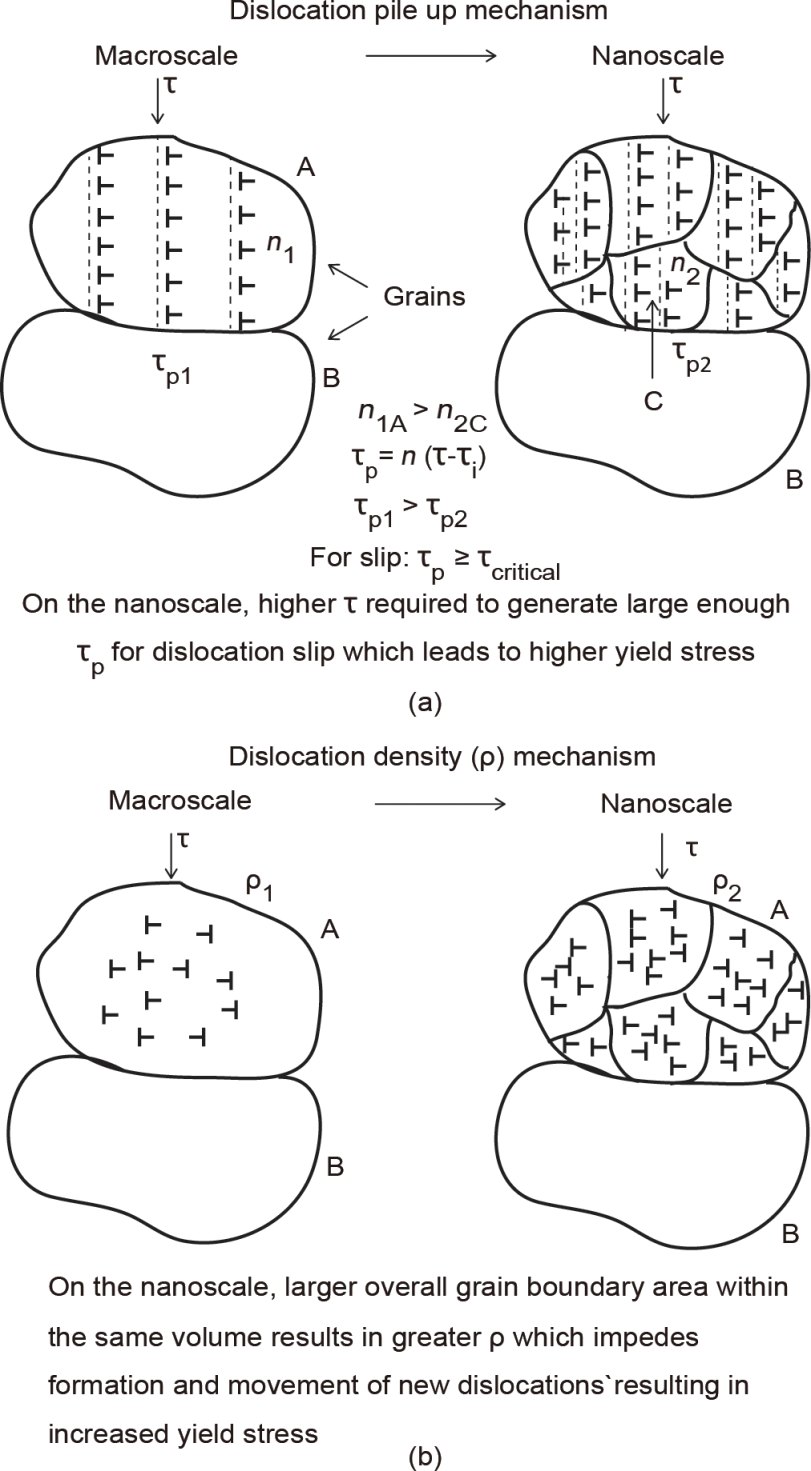
Illustration of (a) Hall–Petch effect by the dislocation pile-up mechanism, where dislocations pile up against the grain boundary under an applied stress τ. The stress at the boundary τ_p_ (pile up stress) which is a function of τ and τ_i_ (internal stress) of the dislocations is larger for a bigger grain size (A) on the macroscale due to the larger number of dislocations compared to that for a smaller grain size (C) on the nanoscale. The number of dislocations (*n*) within the grain on the nanoscale is smaller compared to the macroscale, requiring a larger τ to create a pile up stress (τ_critical_) high enough for dislocation slip and plastic deformation which results in a higher yield stress. (b) Dislocation density mechanism where there is a larger overall grain boundary area as grains become smaller within the same volume, resulting in a greater density of dislocations which impede the formation and motion of new dislocations to accommodate strain gradients and results in higher yield stress.

As the nanoscale is approached, polycrystalline thin films and nano-objects by virtue of their physical dimensions will be composed of materials that have a nanocrystalline structure, i.e., nanometer size grains; as compared to the coarse grained bulk materials, in which grain sizes can vary from 10–300 µm [35]. In the pile-up mechanism illustrated in Figure 3a, for a given applied stress τ, on the grains illustrated by the vertical arrows, dislocations are generated along slip lines, as depicted by the dashed lines, and eventually pile up against the grain boundary. The stress at the grain boundary is called the pile up stress, given as

[2]
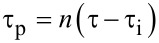


where τ_i_ is the friction stress opposing the movement of the dislocations. The number of dislocations in the pile up in grain A (*n*_1A_) is greater than in grain C (*n*_2C_) due to the larger size which leads to a greater τ_p_. For a slip to occur across the grain boundary, τ_p_ must be greater than the critical stress, τ_critical_. A higher initial τ is therefore required on grain C before the critical stress is reached to allow slip to occur and plastic deformation to continue, which results in a higher yield stress compared to grain A [31–34].

#### Dislocation density mechanism

As mentioned earlier, dislocations can be generated from different sources as shown in Figure 1. As the grain size becomes smaller, shown in Figure 3b, there is a larger overall grain boundary area within the same volume on the nanoscale (right) compared to the macroscale (left), resulting in a greater number of dislocations per unit area or density (ρ). This entanglement of dislocations impedes the formation and motion of new dislocations and multiplication of existing dislocations necessary to accommodate strain gradient and subsequent deformation. This results in greater resistance to deformation and increased yield stress [28,36–37].

## Experimental

### Materials and sample preparation

Si(100) wafers with a native oxide layer (University Wafers, Boston, MA) were ultrasonically cleaned in deionized (DI) water, followed by isopropyl alcohol (IPA) and finally acetone for 15 min each. Polycrystalline Au nanoparticles (Alfa Aesar, Ward Hill, MA) with nominal diameters of 500 nm to be referred to as “Au 500” henceforth, were chosen for the nanoparticle experiments. The 500 nm diameter was the largest size commercially available. It was necessary to use nanoparticles sufficiently larger than the Berkovich indenter tip of radius 100 nm to provide as flat a surface as possible for nanoindentation. This allows for a more accurate determination of the contact area and mechanical properties.

**Figure 4:**
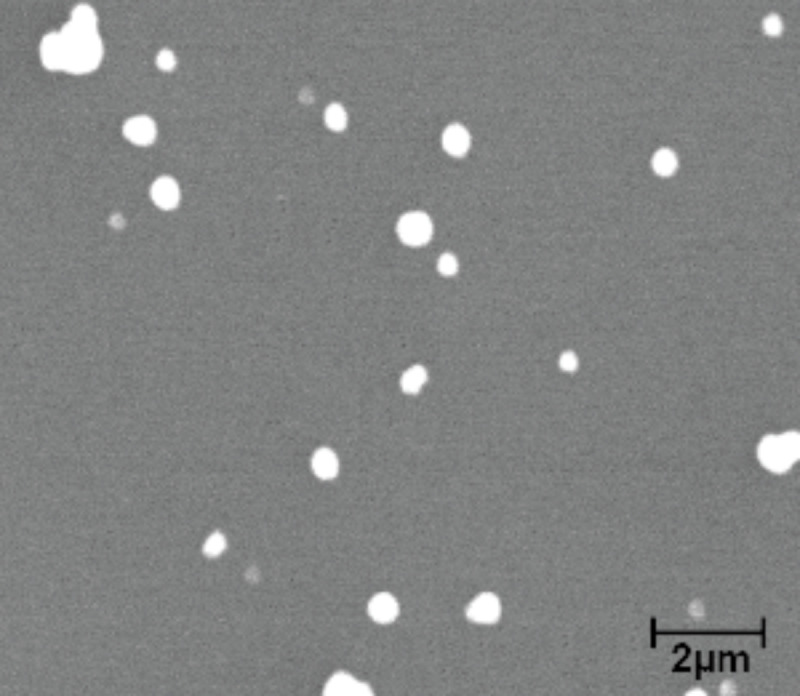
SEM image of spherical Au nanoparticles approximately 500 nm in diameter which are referred to as Au 500.

Figure 4 shows a scanning electron microscopy (SEM) image (S-4300 SEM, Hitachi HTA Inc., Pleasanton, CA) of the nanoparticles. For the nanoparticle experiments conducted, several droplets of Au nanoparticles suspended in DI water were deposited onto the clean Si(100) substrates by using a syringe. A solution concentration of 0.01 mg/mL was used. The substrate was then placed on a hot plate and heated to a temperature of about 70–80 °C and left until the water was evaporated.

**Figure 5:**
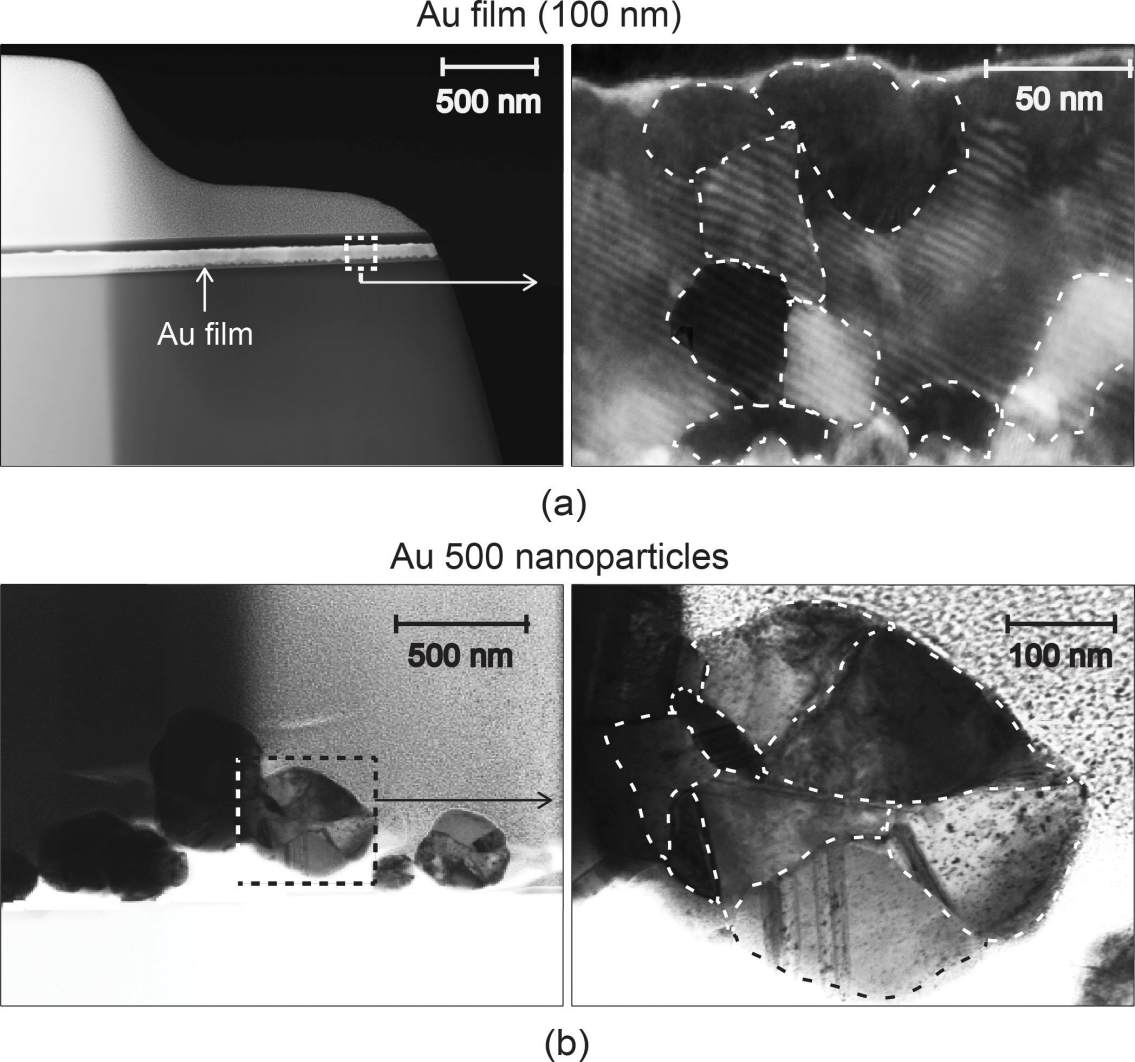
TEM images showing (a) Au film (100 nm) (left) with a magnified view of the section highlighted by the dashed lines shown on the right. In the magnified view, the dashed lines are used to highlight the grains within the film which have an average diameter of 40 ± 9 nm, and (b) several Au 500 nanoparticles (left) with a magnified view of a single nanoparticle with the grains highlighted by the dashed lines (right). The average grain diameter was found to be 96 ± 30 nm.

For thin film experiments, a polycrystalline Au film of approximately 100 nm thickness was deposited onto the surface of the Si(100) substrate by thermal evaporation at an operating pressure of about 0.001 Pa, substrate temperature of 100 °C at a rate of approximately 0.4 nm/s. To observe the grains within the Au film and nanoparticles, focused ion beam (FIB) milling and transmission electron microscopy (TEM) were employed. Cross-sections of samples were cut out by FIB milling (Nova NanoLab 600, FEI, Hillsboro, OR) by using a Ga^+^ ion beam accelerated at a voltage of 30 kV with currents ranging from 0.03 to 28 nA. A Pt coating was deposited on both sets of samples to protect the surfaces during milling. The cross-sections were then lifted out by using a micro manipulator and placed in a holder and observed using a TEM system (Tecnai F20, FEI, Hillsboro, OR, USA) operated at a voltage of 200 kV with a current of 1 nA. Figure 5 shows typical TEM images of both the Au film and Au nanoparticles. Figure 5a shows the Au film (100 nm) on a silicon substrate (left) with a magnified view of the section highlighted by the dashed lines shown on the right. In the magnified view the dashed lines highlight the grains. Figure 5b shows several Au 500 nanoparticles (left) with a magnified view of a single nanoparticle highlighted by the dashed lines. The view on the right shows grains highlighted by the dashed lines. The average grain diameters were found by first importing the TEM images into an image processing and analysis software (ImageJ, National Institute of Health, Bethesda, MD). Second, the outlines of the grains were traced and the enclosed area was determined. The outlines were visible due to the difference in color from one grain to the next as a result of the misalignment of atoms at the grain boundary. Using the area, the diameter of a circle of equivalent area is found and this is taken as the grain diameter. The average grain diameters from the representative images were found to be 40 ± 9 nm for the film and 96 ± 30 nm for the nanoparticle.

### Nanomechanical characterization

#### Nanoindentation

**Figure 6:**
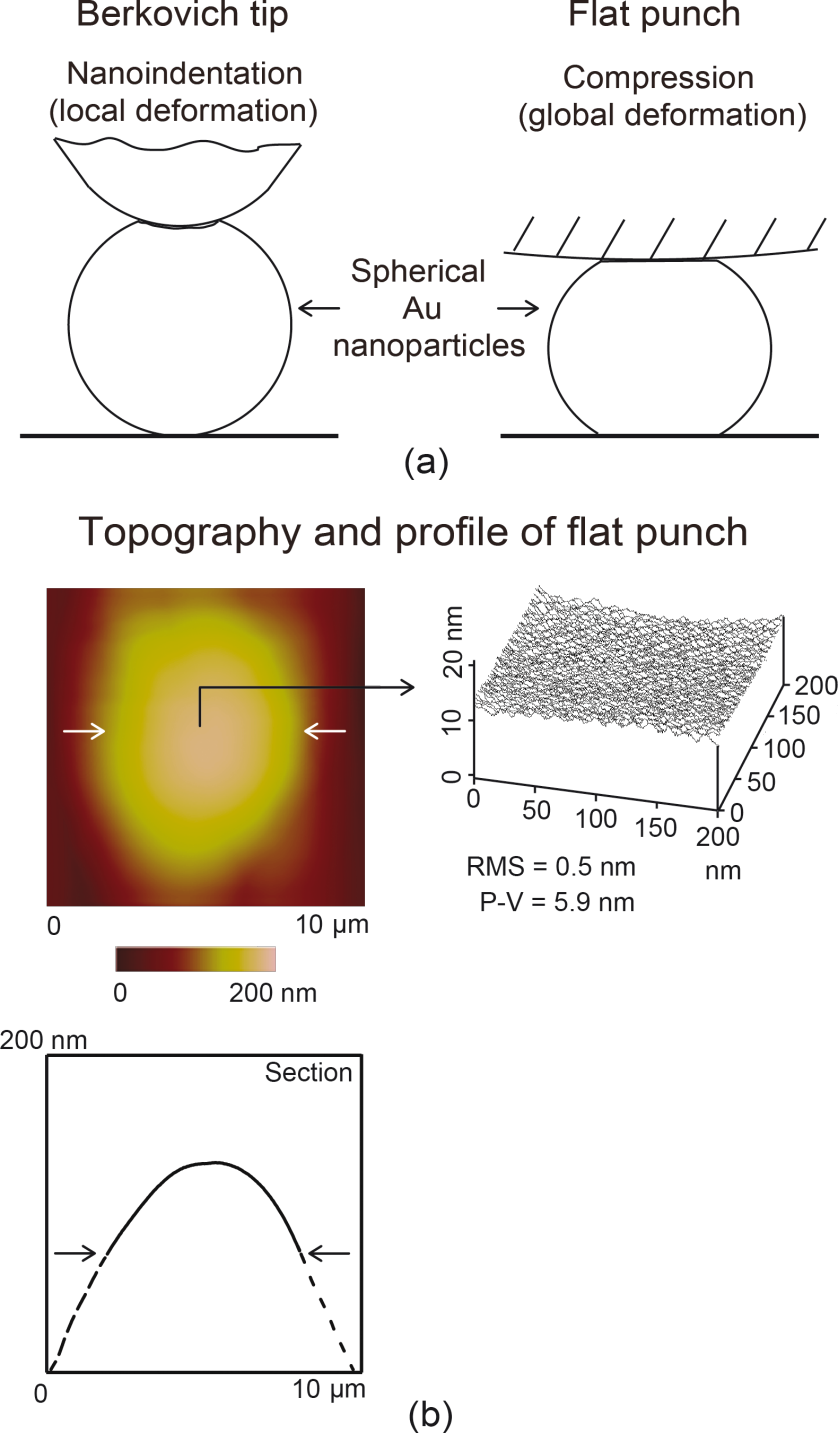
(a) Schematic showing method of deformation by using a Berkovich tip for nanoindentation (local deformation) and flat punch for compression (global deformation) with Au nanoparticles. (b)Topography map (top) and 2-D profile (bottom) of flat punch at the section shown by the pairs of horizontal arrows along with a typical line plot (right) of a 200 nm × 200 nm section indicated by the single horizontal arrow.

All experiments were carried out by using a probe-based scanning nanoindenter head TS 75 Trboscope, (Hysitron, Inc., Minneapolis, MN) attached to an AFM (Bruker Dimension 3100, Santa Barbara, CA) with a diamond tip. For nanoindentation experiments a three-sided diamond pyramidal Berkovich tip of approximately 100 nm in radius was used as shown in Figure 6a (left). Hardness and elastic modulus were obtained as a function of contact depth for the 100 nm thin film by indenting at maximum loads of 20, 40, 60 and 80 µN. The 20 µN load was the lowest load that was possible to produce well defined load–displacement curves for analysis. The 80 µN load was the maximum load possible without the substrate influencing the values of hardness. Effects of creep were studied at 40 and 80 µN by using a hold period of 200 s at the maximum loads. Strain rate effects were investigated at a load of 80 µN for loading and unloading times of 20 and 200 s. The creep and strain rate data gives an indication of whether the experiments are sensitive to hold times and loading–unloading rates and helps to further characterize material behavior which is important for determining suitability for various applications.

Au 500 nanoparticles were indented at maximum loads of 20, 40, 60 and 80 µN similar to the thin film. The Oliver and Pharr [38] method was used to obtain the hardness and the elastic modulus. By using this method the Young’s modulus of elasticity and Poisson’s ratio for diamond were taken as 1140 GPa and 0.07, respectively. Poisson’s ratio for Au was taken as 0.42. The data from these experiments is the average of five measurements on five different nanoparticles for each load. For each Au nanoparticle, further indentation experiments were carried out at intermediate and high loads. These loads were 500 and 1000 µN. Intermediate loads are defined as loads that allow for indents to approximately half the nanoparticle height or more, without fracturing or crushing the nanoparticle. High loads are defined as loads that crush or fracture the nanoparticle. These loads were selected to understand how the nanoparticle deforms under various loading conditions. The duration for loading and unloading was 20 s for all experiments (unless otherwise stated) to prevent the nanoparticle from slipping under the indenter during more rapid and unstable loading. Topography images were also taken before and after indentation with the same tip used for indentation. All experiments were performed at room temperature (23 °C) and 50–55% relative humidity.

#### Compression

For compression experiments a spherical diamond tip of approximately 3.5 µm in radius was used as shown in Figure 6a (right). This can be considered to be a flat punch due to the large radius of the diamond tip compared to the nanoparticles. Figure 6b shows the topography map (top left) and corresponding 2-D profile (bottom left) of the flat punch. The pairs of arrows indicate the section on which the profile is taken. The dashed lines represent the sides of the holder on which the tip is glued. The single arrow points to a representative 200 nm × 200 nm section on the punch that is illustrated by the 3-D map (right). The root mean squared roughness (RMS) is 0.5 nm and the peak to valley (P-V) roughness is 5.9 nm. The low roughness allows for an overall compression of the nanoparticles without indentation due to any large asperities that may be present on the surface.

Three different maximum loads were applied to the nanoparticles. The lowest load for all three cases was 80 µN similar to indentation. This was done to compare the deformation between the two methods. The intermediate and high loads were 1000 and 1500 µN. These loads are similarly defined as those used in indentation. Repeated compression loading experiments, during which several loads are applied to a single nanoparticle, were also performed. Experiments were carried out to explore strain hardening effects on the nanoscale as well as pop-in behavior. The range was 50–250 µN and loads were applied in increasing increments of 50 µN to obtain enough load–displacement curves to clearly observe strain hardening. The range was limited by the nanoparticle either being pushed during imaging or stuck to the diamond tip during compression. This makes imaging and location of the nanoparticle impossible for further compression. The duration for loading and unloading was 20 s for all experiments similar to nanoindentation. Topography images were also taken before and after indentation with the same tip used for compression. To ensure repeatability, each experiment was performed five times and representative data are shown in the results section. All experiments were performed at room temperature (23 °C) and 50–55% relative humidity.

#### Macromechanical characterization

For comparison to the macroscale, data from polycrystalline bulk Au was used from experiments presented by Lozinskii [39]. The Vickers hardness was obtained by using a four sided diamond tip under a load of 1 kg. Typical samples were disc shaped with a diameter of 14.8 mm and thickness of 5 mm. The Young’s modulus for bulk was obtained through resonance of transverse vibrations of a cylindrical specimen which was typically 100 mm in length and 6–8 mm in diameter [39].

## Results and Discussion

In this section, first, hardness, creep and strain rate data are shown for a thin Au film (100 nm). Next, results for nanoindentation using a Berkovich tip and compression using a flat punch are given for Au nano-objects. For both deformation methods, representative load–displacement curves are presented for low, intermediate and high loads. Morphological characterization, before and after deformation, is also presented. Finally load–displacement curves for repeat compression tests using the flat punch are presented.

### Nanoindentation with a Berkovich tip – Indentation of thin films

**Figure 7:**
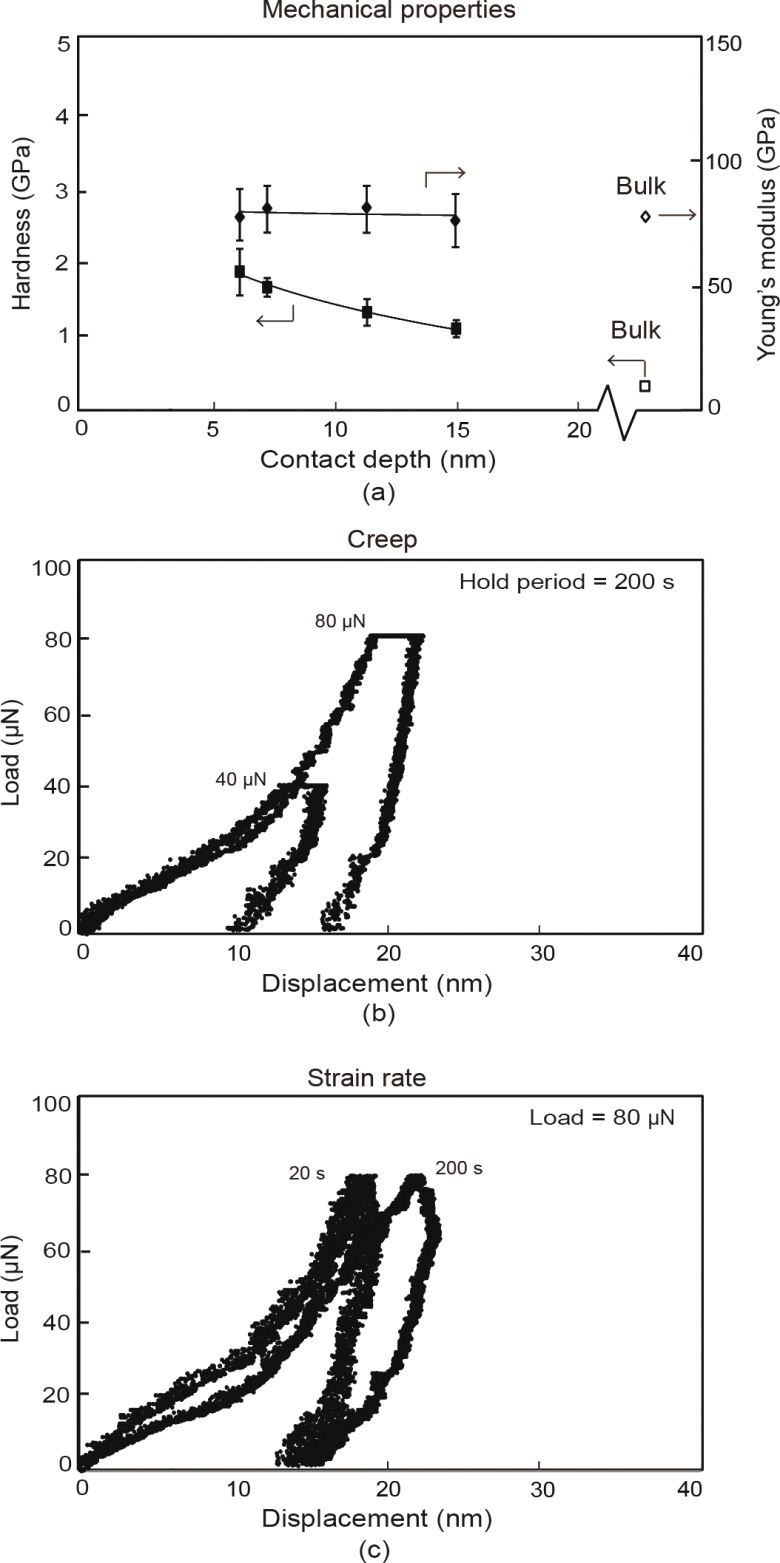
(a) Mechanical properties of thin films with hardness and Young’s modulus as a function of contact depth represented by bold square and diamond datum points, respectively, with corresponding open diamond and square datum points representing bulk mechanical properties. Typical load displacement curves showing (b) creep at maximum loads of 40 and 80 µN with a hold period of 200 s, and (c) effects of varying strain rate at a maximum load of 80 µN for loading–unloading periods of 20 and 200 s.

Indentation with a Berkovich tip was used to determine the mechanical properties and investigate the creep and strain rate behavior of a 100 nm thick Au film as shown in Figure 7.

In Figure 7a the mechanical properties for the thin film are illustrated by the bold diamond and squares, while the open diamonds and squares represent the properties of the bulk material. The hardness and Young’s modulus for the thin film were found at maximum loads of 20, 40, 60, and 80 µN. The Young’s modulus, as a function of the contact depth, is constant for the thin film and similar to that of bulk with little variation. The thin film hardness is greater than that of bulk, which is not believed to be due to the hardness of the substrate. It is generally accepted that the substrate affects the hardness if the depth of penetration is greater than 30% [1] and this limit is not exceeded. Figure 7a shows an ISE where the hardness is greater for shallower penetration depths. This effect was also observed by Bhushan et al. [11] and other researchers as outlined in Table 1. A detailed analysis of the phenomenon was performed by Nix and Gao [30] and explained. As discussed in section Mechanisms, the GNDs along with dislocations that are formed in the absence of strain gradients, known as statistically stored dislocations (SSD), hinder the formation and movement of new dislocations [28–30]. This results in a hardening effect and accounts for the observed increase in hardness at shallower contact depths for the Au film. The higher hardness compared to bulk also has a contribution from the Hall–Petch effect. In this case an increase in yield stress or hardening is observed compared to the macroscale due to the smaller grain sizes inherent in the thin film compared to bulk. The grain diameters in the representative image for the film shown in Figure 5a were found to be 40 ± 9 nm. The hardening can occur through dislocation density mechanism and pile-up mechanism outlined in section Mechanisms and illustrated in Figure 3a,b. However, due to the submicron thickness of the film, the nanosized grains limit the number of dislocation pile-ups. It is believed the dislocation density mechanism is primarily responsible for the higher hardness.

Figure 7b shows creep for typical load–displacement curves with maximum loads of 40 and 80 µN with a hold period of 200 s. Figure 7c shows indentation–displacement curves for different strain rates with a maximum load of 80 µN for loading and unloading times of 20 and 200 s. As mentioned in section Experimental, the creep and strain rate data gives an indication of whether the experiments are sensitive to hold times and loading and unloading rates. For the creep data there is there is very little difference in the displacement during the holding times at a load of 40 µN compared to 80 µN. The strain rate data also shows a small amount of displacement from a loading and unloading time of 20–200 s. Both experiments were performed at room temperature. Since Au is inert and does not form an oxide layer it is not believed that the increased displacement at longer holding and loading times is caused by a contaminant layer. According to analysis of experimental data from several research groups by Li et al. [40], creep can occur in most materials, even at room temperature. In materials with small grain sizes (smaller than 0.3–0.4 µm) indentation creep is dominated by grain boundary (cobble) diffusive creep, which occurs by addition or removal of atoms from the boundary between two grains [40–41]. This diffusional creep is believed to be responsible for increased displacements observed for creep and strain rate data. The low sensitivity of Au to creep and strain rate is useful in tribological applications on the nanoscale in situations where constant load or varying loading rates occur. This prevents the film from deforming easily.

Similar experiments were not performed on the Au 500 nanoparticles since over longer hold and loading and unloading times the possibility of nanoparticles rotating and sliding is increased and results in the nanoparticles slipping out from under the indenter.

### Nanoindentation with a Berkovich tip – Localized deformation on Au nanoparticles

**Figure 8:**
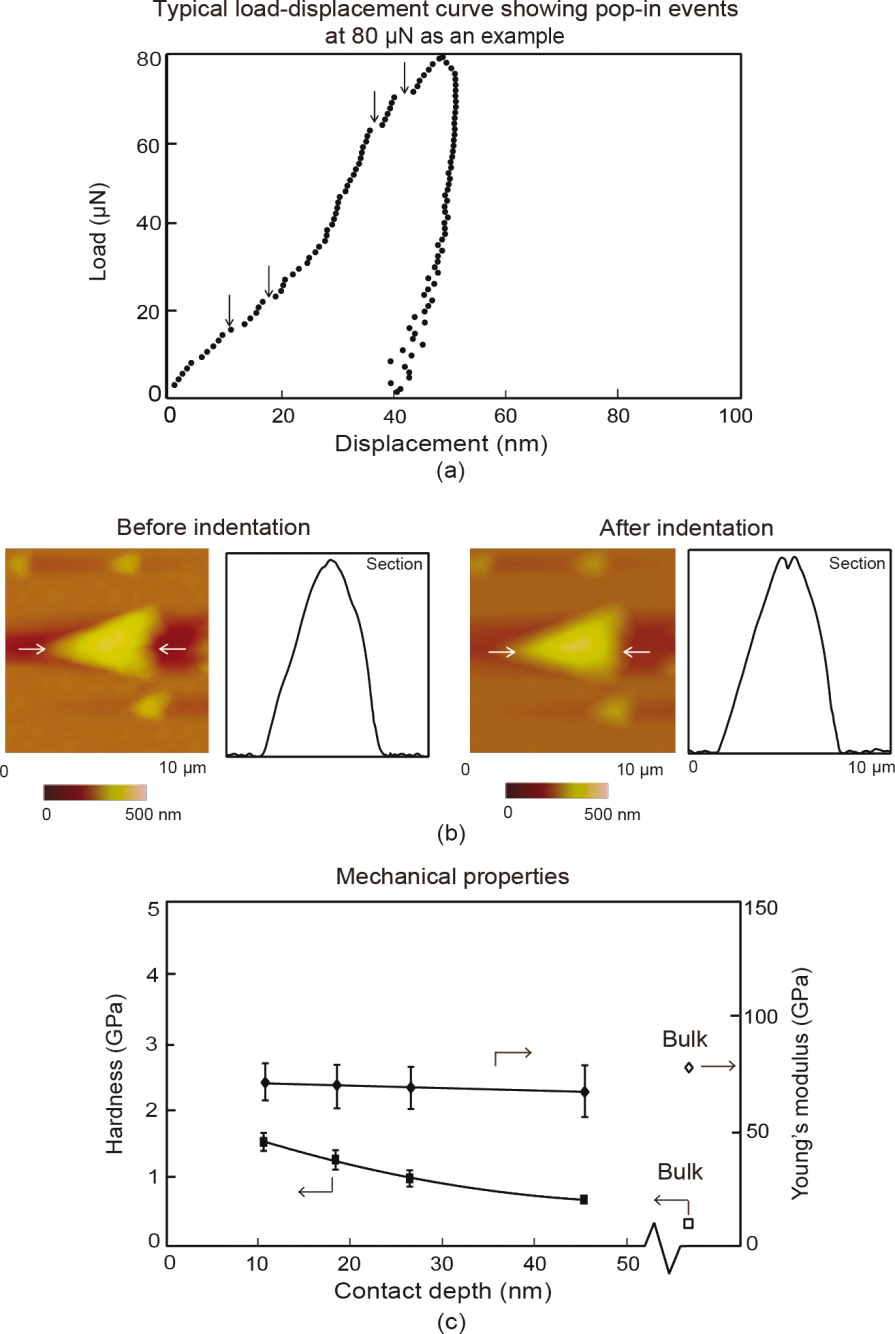
(a) Typical load displacement indentation curve at a maximum load of 80 µN with vertical arrows showing pop-in events and (b) topography maps and 2-D profiles at sections shown by the horizontal arrows before indentation and after indentation. (c) Mechanical properties of Au 500 nanoparticles with hardness and Young’s modulus as a function of contact depth represented by bold square and diamond datum points, respectively, with corresponding open diamond and square datum points representing bulk mechanical properties.

For probing of mechanical properties of the polycrystalline Au 500 nanoparticles, indentation was performed at a maximum load of 80 µN. Figure 8 shows a typical load–displacement curve for indents at a maximum load of 80 µN, with topography maps of the nanoparticles over a 10 µm × 10 µm scan area and 2-D profiles before and after indentation. The topography maps of the nanoparticles appear not totally circular due to tip shape convolution effects, however they are used to confirm the indentation of the nanoparticle and that the nanoparticle did not slip during loading and unloading. The vertical arrows on the load–displacement curves point to pop-in events during indentation. The horizontal white arrows indicate the nanoparticle of interest along with the section on which the profiles were taken. The pop-in events correspond to generation of new dislocations and multiplication of existing dislocations within the grain boundaries, which leads to an increase in displacement at a constant load. The eventual hardening is due to the dislocation density mechanism and possibly the pile-up mechanism, as discussed in section Mechanisms. Similar to the thin film, it is believed that the submicron size of the Au nanoparticles, limits the number of pile-ups and the dislocation density mechanism is primarily responsible for the increase in hardness. This process repeats and results in subsequent slip and generation and multiplication of dislocations in a neighboring grains [42–44] resulting in further pop-ins. Figure 8c shows the mechanical properties for the Au 500 nanoparticles as illustrated by the bold diamond and squares, while the open diamonds and squares represent the properties of the bulk material. The hardness and Young’s modulus for nanoparticles were found at maximum loads of 20, 40 60, and 80 µN. The Young’s modulus, as a function of contact depth, is constant for the nanoparticles and slightly lower than that of bulk with little variation. An ISE for the hardness is also observed, similar to the thin film due to higher strain gradients at shallower depths. The hardness is also higher for the nanoparticles compared to bulk Au. This follows the ‘smaller is stronger’ phenomenon. The average grain diameter for the nanoparticle as shown in Figure 5b was found to be 96 ± 30 nm. It is believed that the nanometer-sized grains are responsible for enhanced hardness. This is based on the mechanisms described in the Hall–Petch effect, for which the dislocation density mechanism provides a greater contribution to hardness than the pile-up mechanism. The decreasing grain sizes leads to higher yield stress and results in an increased hardness as observed.

Table 2 presents data for hardness and Young’s modulus of elasticity, in addition to the contact depth during indentation at a representative load of 80 µN for the Au nanoparticles and film. The hardness of the nanoparticles was found to be lower than the thin film. This is expected since the film thickness of 100 nm is less than the diameter of Au 500 and has smaller grains with diameters of 40 ± 9 nm compared to 96 ± 30 nm and higher resistance to yield. Both scales (100 nm and 500 nm) show a higher hardness compared to bulk. This expands the possible uses of nanoscale Au in harsh environments where resistance to deformation under loading is important for reduced friction and wear.

**Table 2:** Nanomechanical properties of Au nanoparticles and thin film at a maximum load of 80 µN compared to bulk Au.

	nanoparticle diameter/ Film thickness (nm)	contact depth (nm)	hardness (GPa)	Young’s modulus (GPa)	explanation for enhanced hardness

Au 500	513 ± 38	45 ± 4	0.7 ± 0.1	70 ± 11	Hall–Petch, strain gradient plasticity
Au thin film (100 nm)	100	15 ± 1	1 ± 0.1	76 ± 11	Hall–Petch, strain gradient plasticity
Bulk Au [46]			0.22	78	

**Figure 9:**
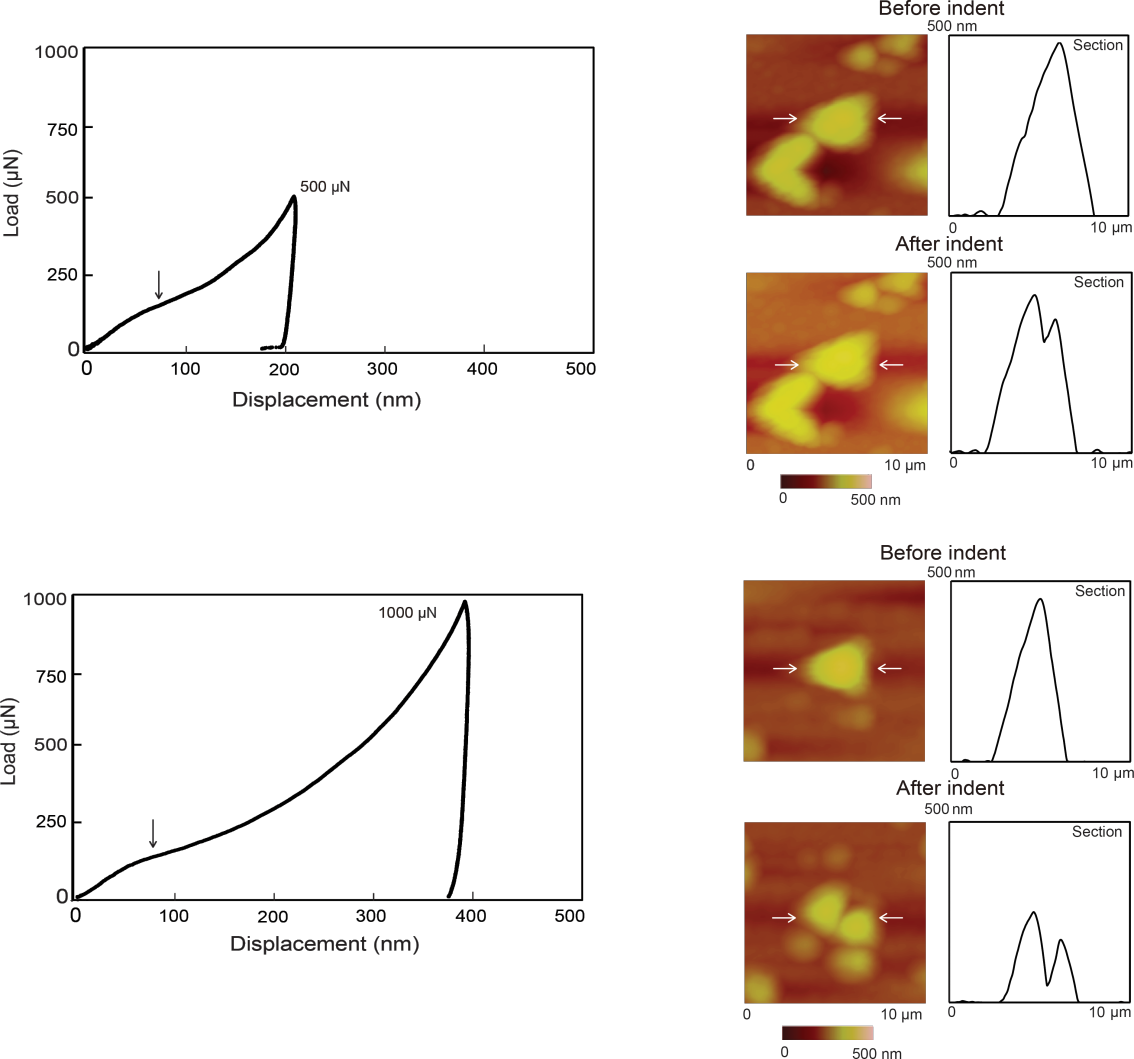
Load displacement curves for intermediate loads 500 µN and high loads 1000 µN for Au 500 with the vertical arrows depicting slip events (left), and topography maps and 2-D profiles, at sections indicated by the horizontal arrows before indentation (first row) and after indentation (second row) (right).

Figure 9 shows examples of load–displacement curves at intermediate and high loads (left) along with topography maps of the nanoparticles over a 10 µm × 10 µm scan area and 2-D profiles before and after indentation (right). The intermediate and high loads were 500 and 1000 µN, respectively. The horizontal white arrows in the topography maps indicate the nanoparticle of interest along with the section on which the profiles were taken. The vertical arrows shown in the load–displacement curves (left) point to slip events where the nanoparticles start to break apart during indentation leading to displacement of material below the Berkovich tip.

### Compression with a flat punch – Deformation of entire Au nanoparticle

**Figure 10:**
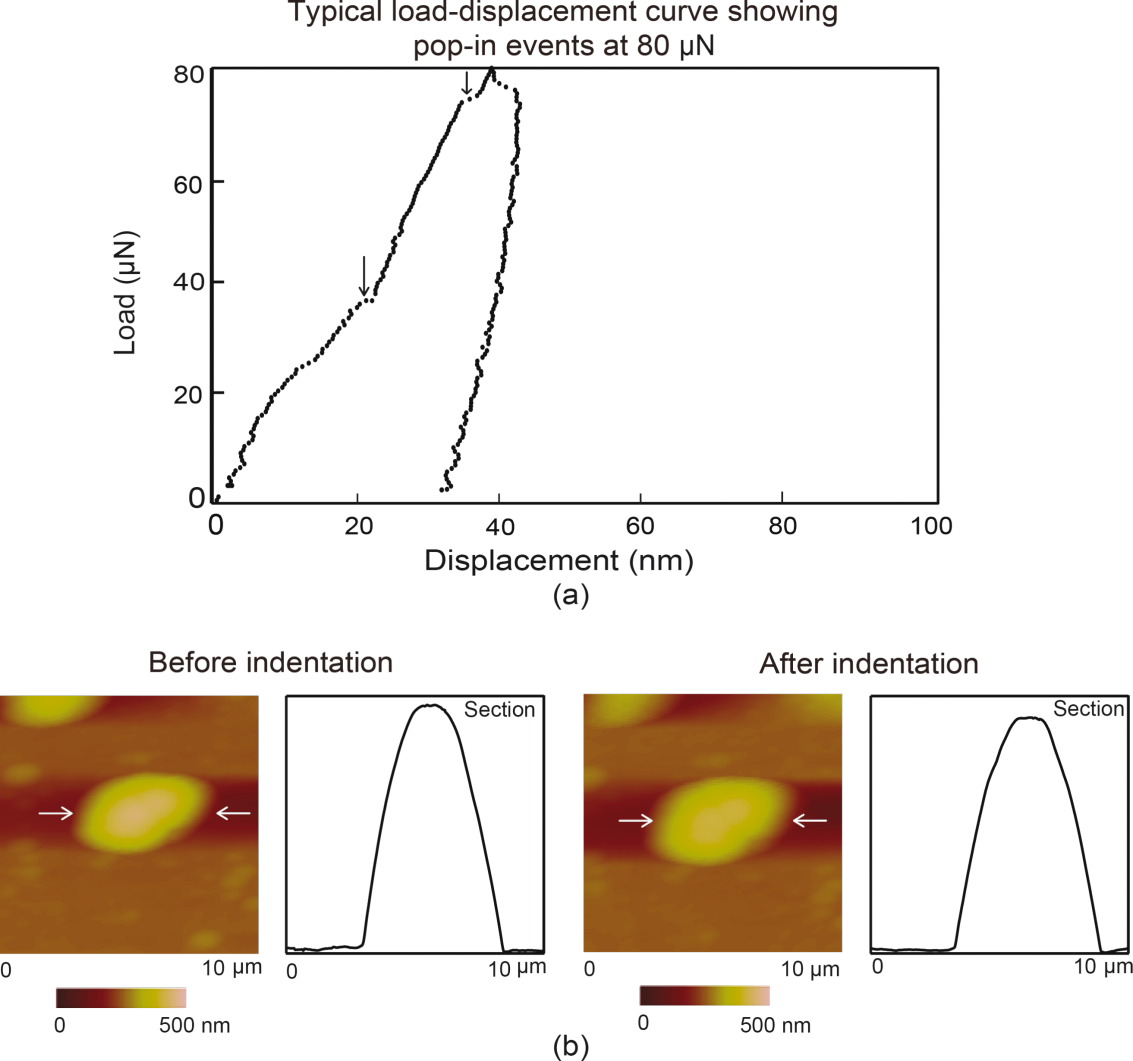
(a) Typical load displacement compression curve at a maximum load of 80 µN for Au 500 with vertical arrows showing pop-in events and (b) topography maps and 2-D profiles at sections shown by the arrows before compression (first row) and after compression (second row).

Nanoparticles were compressed to examine the differences between local deformation (nanoindentation) and global deformation (compression). Compression tests, as well as indentation tests, simulate the types of contacts nanoparticles encounter during different friction and wear conditions. For this purpose, a tip approximately 3.5 µm in radius was used to carry out compression tests. Figure 10 shows a typical load displacement curve for compression at a maximum load of 80 µN, along with topography maps of the nanoparticles over a 10 µm × 10 µm scan area and 2-D profiles before and after compression. The topography maps of the nanoparticles appear not completely circular because of tip-shape convolution effects, however, they are used to confirm the compression of the nanoparticles. The vertical arrows point to pop-in events during indentation. In Figure 10b the horizontal white arrows indicate the nanoparticle of interest along with the section on which the profiles were taken. Pop-in events due to dislocations were observed as with nanoindentation. These occur in the latter half of the loading curve unlike with indentation, which shows pop-ins throughout the loading curve. Larger contact area of the flat punch compared to the sharp tip for nanoindentation results in a lower contact pressure. During the early stages of loading, the low pressure does not generate a sufficient internal stress for dislocation nucleation, multiplication and slip to occur which prevents a sudden displacement of material or pop-in.

**Figure 11:**
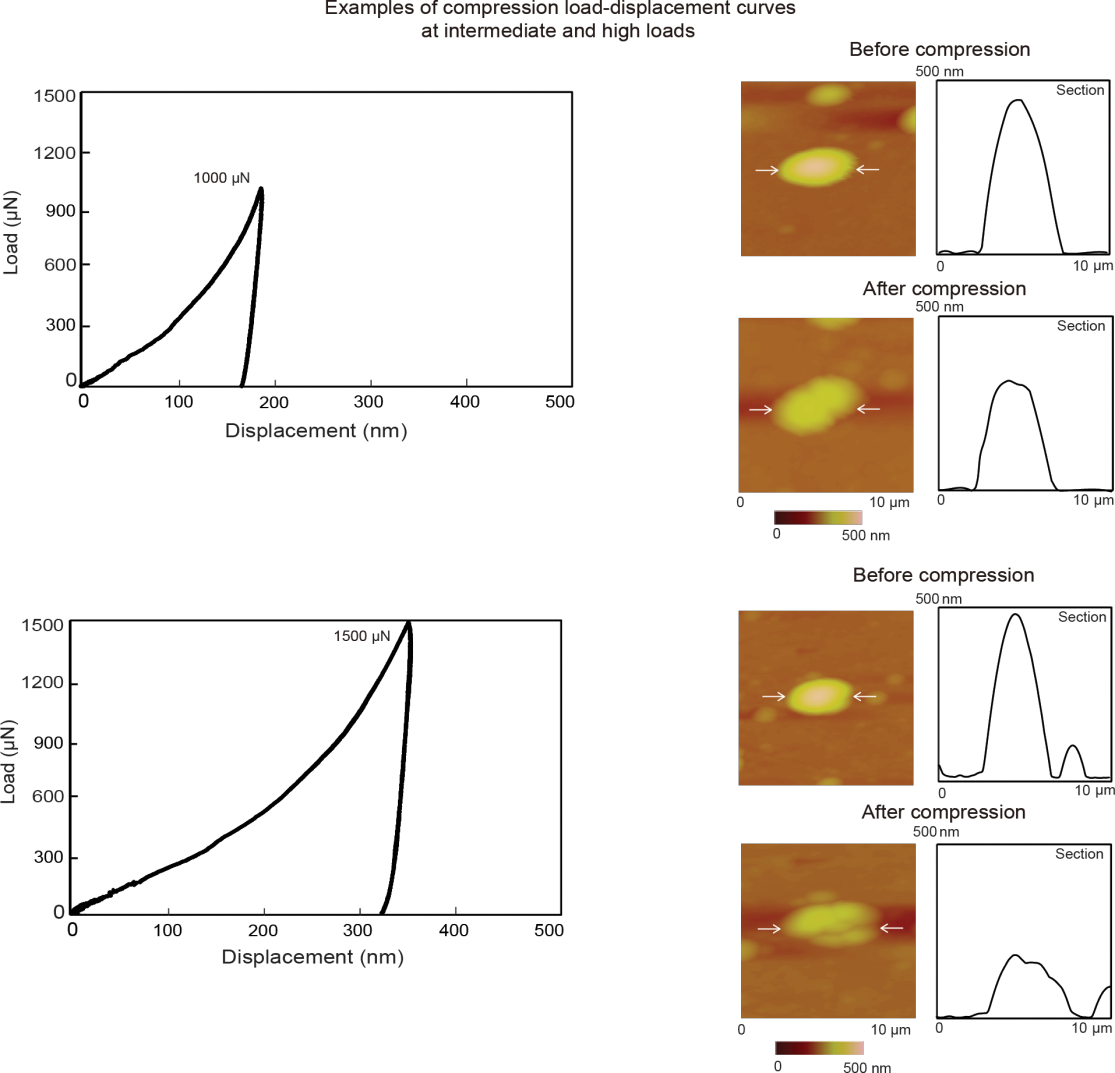
Load–displacement curves for intermediate loads 1000 µN and high loads 1500 µN with topography maps and 2-D profiles at sections shown by the arrows, before compression (first row) and after compression (second row).

Figure 11 shows examples of load–displacement curves at intermediate and high loads (left) along with along with 2-D topography maps of the nanoparticles over a 10 µm × 10 µm scan area and profiles before and after compression (right). The intermediate and high loads were 1000 and 1500 µN. The horizontal white arrows in the topography maps indicate the nanoparticle of interest along with the section on which the profiles were taken. No slip events were observed as during nanoindentation. In this case the entire volume of the nanoparticle is being compressed and material does not slip out from under the flat punch.

**Figure 12:**
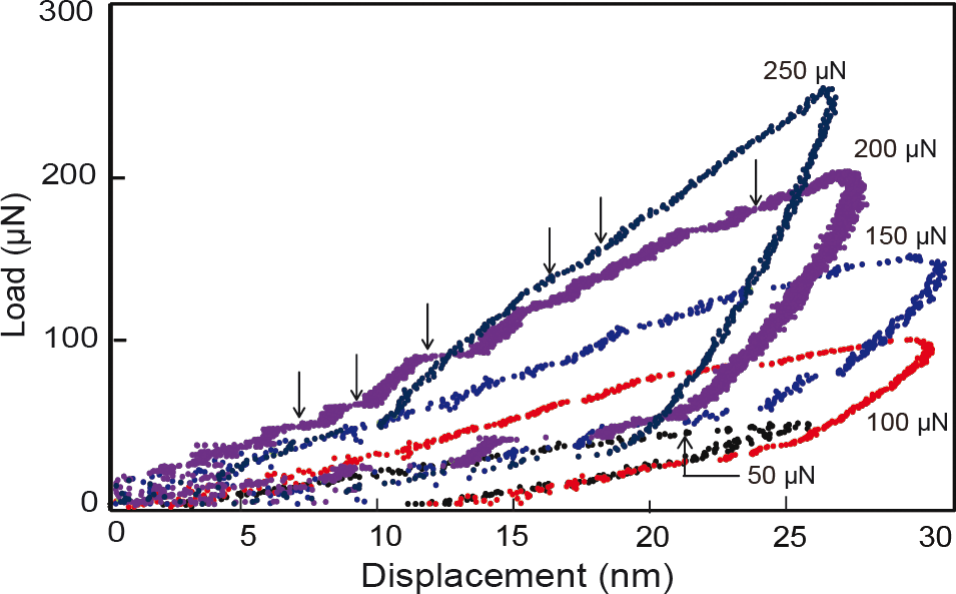
Examples of repeat load–displacement curves for Au 500 nanoparticles with the corresponding maximum loads for each compression event. Vertical arrows point to pop-in events. Increments of 50 µN were applied in a range of 50–250 µN. Evidence of strain hardening can be seen where successive loads after 150 µN result in the displacement being the same or less after unloading.

Repeated compression test were also performed with increasing loads. This provides an opportunity to study strain hardening on the nanoscale and to further investigate pop-in behavior as the loads are increased. Figure 12 shows load–displacement curves for repeated loads for the nanoparticles. Loads were applied with increments of 50 µN for a range 50–250 µN. Initially as the load increases the displacement increases until a load of 150 µN. For the 200 and 250 µN loads, the displacements are almost the same as the 50 µN load and less than the 100 and 250 µN loads. This indicates a hardening effect. Higher loads were not possible as the nanoparticles would either adhere to the indenter tip or slip out, resulting in the nanoparticle not being found during subsequent imaging. It is believed that the dislocations generated either continue to pile up or the already existing dislocations created during the previous loading phase prevents the movement of new dislocations resulting in strain hardening. A larger number of pop-in events were observed especially at 200 and 250 µN which would indicate that the high stress generated by the accumulated dislocations from previous compressions along with newly formed dislocations results in multiple slip events during loading. The hardening observed with the repeated compression can be useful in situations in which repeated contacts with surfaces occur such as tribological systems on the macro- to nanoscale.

## Conclusion

Au nanoparticles 500 nm in diameter along with a 100 nm thick film were investigated to determine their mechanical properties on the nanoscale and to investigate scale effects. Nano-object studies provided the opportunity to compare local deformation (nanoindentation) with a sharp tip and global deformation (compression) with a flat punch by using a nanoindenter. This was performed under three loading regimes, described as low, intermediate and high. Strain hardening compression was also performed by repeated loading. For the thin film, creep and strain rate behavior was also investigated.

For indentation with a sharp tip, an indentation size effect (ISE) was observed and the hardness of Au 500 and Au film increased due to higher strain gradients at shallower penetration depths. The hardness of the film was higher than that of the nanoparticles with both being higher than that of the bulk because of the Hall–Petch effect, which was explained by using the dislocation density mechanism and the pile-up mechanism with a greater contribution to the hardness coming from the dislocation density mechanism. TEM analysis confirmed the smaller grain sizes of the film compared to the nanoparticles. The large strain gradients also contributed to an increased hardness compared to bulk Au. Load displacement curves for Au 500 at low loads revealed pop-in effects, which occur due to generation and slip of dislocations. For the thin film, creep and strain rate tests showed displacements of a few nanometers for the hold period and increased loading–unloading times. This is believed to be due to diffusion creep associated with grain boundary translation.

For compression pop-in effects from the sudden displacement of material as a result of accumulation and slip of dislocations, were observed during loading similar to nanoindentation. Repeat compression tests showed a strain hardening effect with each subsequent load. The resulting displacement at each new load was either the same or lower than the previous. This was due to increased resistance to deformation as a result of a greater density of dislocations restricting the creation and movement of new dislocations being formed. Several pop-in effects were observed during repeat compression tests at increasing loads due to accumulation of dislocations from previous loads and formation of new dislocations.

Further studies would include characterizing the sub-micron structure of the film and nanoparticles in terms of grain size and dislocation content and behavior. This would give a more precise determination of the relative contributions of dislocation mechanisms responsible for enhanced hardness. The knowledge gained will have far reaching effects when designing macro- to nanoscale systems that incorporate materials with nano-dimensions.
